# Functionalized Multi‐Walled Carbon Nanotube Enhanced Myogenic Differentiation for Aligned Topography‐Induced Skeletal Muscle Engineering

**DOI:** 10.1002/smll.202504992

**Published:** 2025-07-24

**Authors:** Tianqi Feng, Ludovica Ceroni, Lisa Eveline Tromp, Clio Siebenmorgen, Stefano Casalini, Enzo Menna, Patrick van Rijn

**Affiliations:** ^1^ University of Groningen University Medical Center Groningen Deusinglaan 1 Groningen 9713 AV The Netherlands; ^2^ Department of Chemical Sciences University of Padua & INSTM Via Marzolo 1 Padova 35131 Italy

**Keywords:** coating, electrochemical impedance spectroscopy, multi‐walled carbon nanotube, muscle tissue engineering, myoblasts, topography

## Abstract

Skeletal muscle engineering utilizing bio‐activators and myogenic cells to regenerate tissues for volumetric muscle loss offers a promising alternative to tissue grafts. Modified biointerfaces with aligned micro‐scale topography and electroconductivity are critical for directing cellular behavior toward functional muscle constructs. This study modified polydimethylsiloxane (PDMS) with aligned surface topography and functionalized multi‐walled carbon nanotubes (fCNTs), creating a conductive scaffold (0.11 µScm^−1^ vs original 0.51 nScm^−1^) with regulated hydrophilicity (76 ± 2° vs original 50 ± 10° in water contact angle) and enhanced protein absorption. The fCNT‐wrinkled surfaces maintained >90% cell viability while promoting aligned myotube formation. Specifically, fCNT integration with aligned topography increased myotube length from 303.74 ± 27.61 µm to 441.63 ± 10.27 µm and elevated fusion index to 40.43% ± 2.67% within three differentiation days. Immunostaining confirmed enhanced myogenic maturation through improved cell alignment and nuclei organization. These biophysical modifications synergistically accelerated myoblast differentiation while maintaining cytocompatibility by combining electrical conductivity, optimized wettability, and directional cues. The demonstrated capacity to physiologically mimic native muscle microenvironments highlights this strategy's potential for improving muscle regeneration therapies through precise control of surface‐electrotopographical properties.

## Graphical abstract

Conductive interfaces are known to support muscle engineering approaches, but without proper guidance, the muscle will not be functional if not properly aligned. Therefore, combining carbon nanotubes and aligned topography enhances muscle tissue engineering approaches, displaying a synergetic effect.

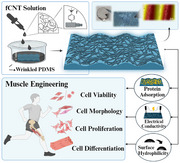

## 1 Introduction

Severe injuries with mass loss of more than 20% of muscle tissue are called volumetric muscle loss disease. It is the traumatic or surgical loss of skeletal muscle due to ineffective endogenous regeneration, fibrosis, and scar formation that results in permanent damage and resultant functional impairment.^[^ ^1^ ^]^ The most common treatment method for severe muscle tissue injury is surgical reconstruction, but its application is strongly limited by its low survival rate, frequent donor lesions, and lack of donor sources.^[^ ^2^ ^]^ Therefore, there is an urgent need for a new and highly effective therapeutic strategy to treat muscle injuries. To date, tissue engineering is a promising strategy to develop functional substitutes for bioactive substances to accelerate damage reconstruction or facilitate tissue regeneration by cultivation or construction in vitro.^[^ ^3^, ^4^ ^]^

Tissue engineering offers a promising therapeutic approach by combining beneficial biomaterial scaffolds, bioactive molecules, and cells to repair or replace damaged tissue.^[^ ^1^ ^]^ Understanding and utilizing the interactions between cells and scaffolds is the main strategy for which several physicochemical approaches can be utilized as physicochemical properties are known to influence amongst others, cell proliferation,^[^ ^5^ ^]^ migration,^[^ ^6^ ^]^ and differentiation^[^ ^7^ ^]^ in general, but also for skeletal muscle,^[^ ^7^, ^10^ ^]^ specifically, skeletal muscle presents a highly organized structure with extended parallel fascicles of multinuclear muscle tubes.^[^ ^7^, ^8^, ^11^ ^]^ However, improper alignment of muscle tubes can also lead to ineffective transmission and contractility of functional fiber regeneration.^[^ ^9^ ^]^ In this case, the orientation of myotube formation in muscle engineering applications needs to be considered. Previous studies showed the importance of considering the natural topographic structure of skeletal muscle tissue to design scaffolds which the size and shape of which had been taken into consideration in biomaterial design.^[^ ^7^, ^10^ ^]^

When considering the function of muscle, contraction activity is one of the unique behaviors of muscle tissue, which is in response to electrical signals.^[^ ^1^ ^]^ The synergy between physico‐mechanical transduction and biochemical‐mechanical signaling in biological environments created by the ECM molecular and cellular interactions affects cell behavior, such as cell phenotype, cell movement, and cell differentiation.^[^ ^9^ ^]^ The ECM represents a special electroactive surrounding for cells, mainly composed of differently charged molecules that cooperate with piezoelectric collagen fibers capable of generating electrical signals in response to mechanical stimuli.^[^ ^11^, ^12^ ^]^ Some studies found that electroactive materials could mediate the ion flow and affect the activation of the calcineurin pathway to promote myoblast differentiation, which could provide a promising candidate for muscle tissue engineering scaffolds.^[^ ^12^, ^13^, ^14^ ^]^

Among conductive biomaterials, carbon nanotubes have electrical conductivity similar to natural muscle,^[^ ^15^, ^16^ ^]^ which are promising candidates for scaffolds for muscle tissue engineering. Carbon nanotubes (CNT) are carbon allotropes of the carbon nanostructures (CNS) family that connect to a sheet of graphene, as a CNT can be regarded as graphene rolled into an elongated cylindrical shape. These promising materials based on sp2‐hybridized carbon atoms have a diameter in the nanometre scale (<100 nm) and a variable length of up to several micrometers.^[^ ^17^ ^]^ Moreover, CNT can also be arranged in several concentric cylindrical shells of graphene sheets coaxially placed around a hollow central core, resulting in multi‐walled CNT, which offers excellent electrical signal conduction. Multi‐walled CNTs have already been extensively employed in tissue engineering applications, such as cardiac repair, tissue healing, and bone regeneration, due to their special aspect ratio and excellent electrical as well as thermal conductivity properties.^[^ ^18^ ^]^ Therefore, concerning tissue engineering, Imaninezhad et al. recently demonstrated that the electrostatic fields generated at the interface between non‐excitable cells and an appropriate scaffold can promote cell growth by regulating their membrane potential.^[^ ^19^ ^]^ In particular, the introduction of modified Multi‐walled CNT in a polymeric scaffold such as PLA resulted in an extracellular environment suitable for the proper functionality of fibroblasts.^[^ ^20^ ^]^ Moreover, we have already successfully tested the properties of functionalized Multi‐walled CNT for the fabrication of composite scaffolds of PLA for the differentiation of neuronal cells,^[^ ^21^ ^]^ of PVA for the regeneration of nerve tissue,^[^ ^22^ ^]^ and of alginate for the differentiation of muscle stem cells.^[^ ^23^ ^]^

In tissue engineering, the main strategy of scaffold design mimics the conditions of the natural environment around the cell to produce specific biological effects on cell behavior. Based on the above‐mentioned clues, both topography and electrical signaling play a dominant role in the transfer of mechanical signals between the extracellular and intracellular environments in the modulation of cellular behavior. For this purpose, we are committed to creating a conductive interfacial structure with a specific morphology to investigate the effect of the composite interfacial properties on the formation of skeletal muscle, which could provide a feasible strategy for further research and development of 3D materials.

In this study, a micro‐scale aligned topography was used to regulate cell orientation and is combined with a positively‐charged functionalized multi‐walled CNT (fCNT)‐based coating by using a combination of plasma treatment and dip coating method, forming a uniform wrinkled electroactive substrate.^[^ ^6^ ^]^ The topography used is a wrinkled topography with a wavelength of 10 µm, which was developed and optimized by us previously.^[^ ^8^, ^24^ ^]^ Morphological observation, hydrophilicity, and electrical conductivity were determined to evaluate the efficacy of the coating. Cell biocompatibility and cell proliferation, and differentiation studies were conducted to assess the substrate's capacity to support cellular growth and the ability to promote cell differentiation for muscle formation. Based on these evaluations, the capability of the fCNT‐coated wrinkled PDMS for muscle formation was confirmed, and it is envisioned that the approach to be broadly applicable for scaffold applications, especially for aligned tissue regeneration, such as nerve repair, osteogenesis, and cardiac therapy.

## 2 Results

### 2.1 Fabrication and Characterization of fCNT‐Coated PDMS

To create the CNT‐enhanced surface topography, trimethyl ammonium benzene‐modified multi‐walled carbon nanotubes were used as a positively charged fCNT and were combined with negatively charged PDMS to apply a coating through electrostatic attraction to get a unique nano‐ and micro‐scale topography surface for enhanced myofiber formation and alignment. In our work, the size of the wrinkle pattern we choose is inspired by our previous study.^[^ ^7^, ^25^ ^]^ The wrinkle size could regulate the cell morphology and modulate cell alignment along the groove direction. The wavelength of 9.67 ± 0.32 µm and the amplitude of 1.00 ± 0.08 µm could make the cell elongate well.

**Figure 1:**
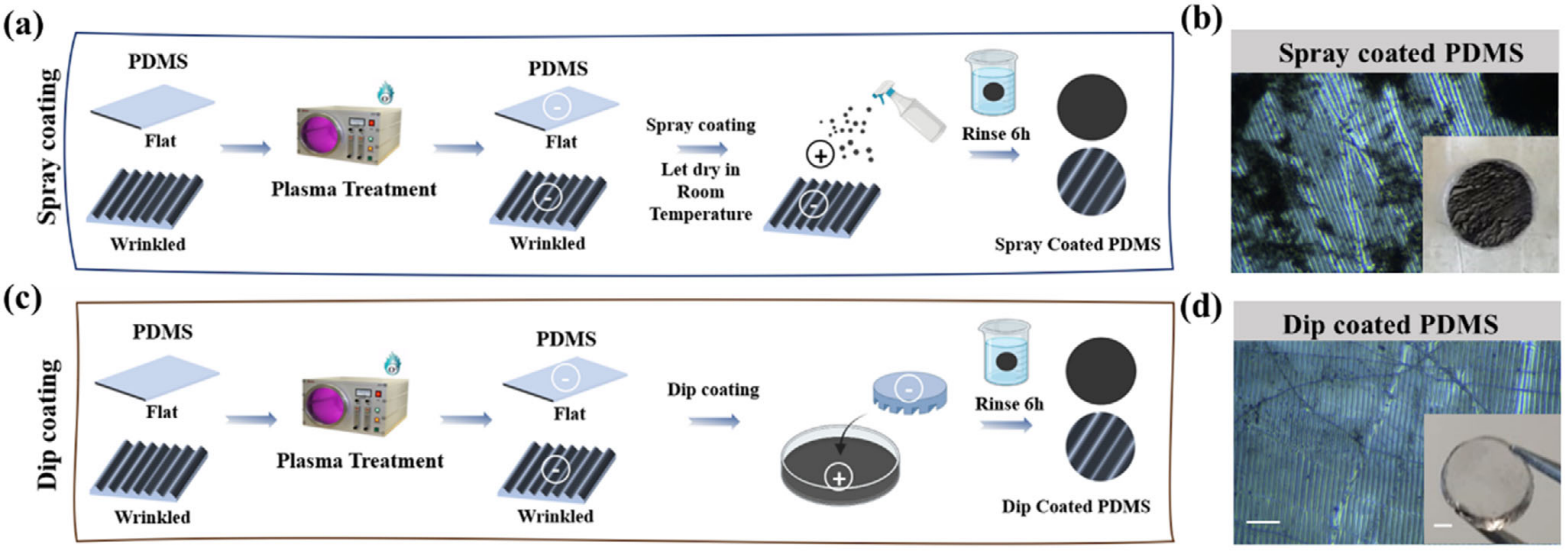
Schematic illustration of the coating methods. After flat and wrinkled PDMS was obtained by following the procedure optimized by our group, the PDMS surface was air‐plasma treated by a plasma oven to obtain a negatively charged PDMS surface. After oxidation, spraying the solution of positively charged fCNT solution on the oxidized PDMS slides to complete the spray‐coated procedure, a) overnight and rinse in water to remove the extra fCNT on the surface to get a spray‐coated sample, b). The dip coating method c) was followed by immersing the oxidized PDMS slides into the solution of positively charged fCNT overnight and washing with water to remove the extra fCNT on the surface to get the dip‐coated sample d). The samples were observed by optical microscope (scale bar, 50 µm) and camera (scale bar, 2 mm).

Two deposition methods were explored: i) spray (**Figure** **1**a,b) and ii) dip coating (Figure 1b–d) for both flat and wrinkled PDMS. The latter approach offered better control over the final layer morphology of fCNTs. More precisely, uncontrolled evaporation of the solvent leads to dark spots (Figure 1b) due to fCNTs aggregation when using the spray coating technique. On the contrary, using dip coating, it was possible to achieve a homogeneous fCNTs layer. (Figure 1d) The dip coating process, combined with the electrostatic attraction of the positively charged fCNT and the negatively charged surface, ensured a very thin and homogenous surface coating without any visible aggregates (Figure 1d). In this case, the dip‐coating method was chosen and applied for the following experiment.

**Figure 2:**
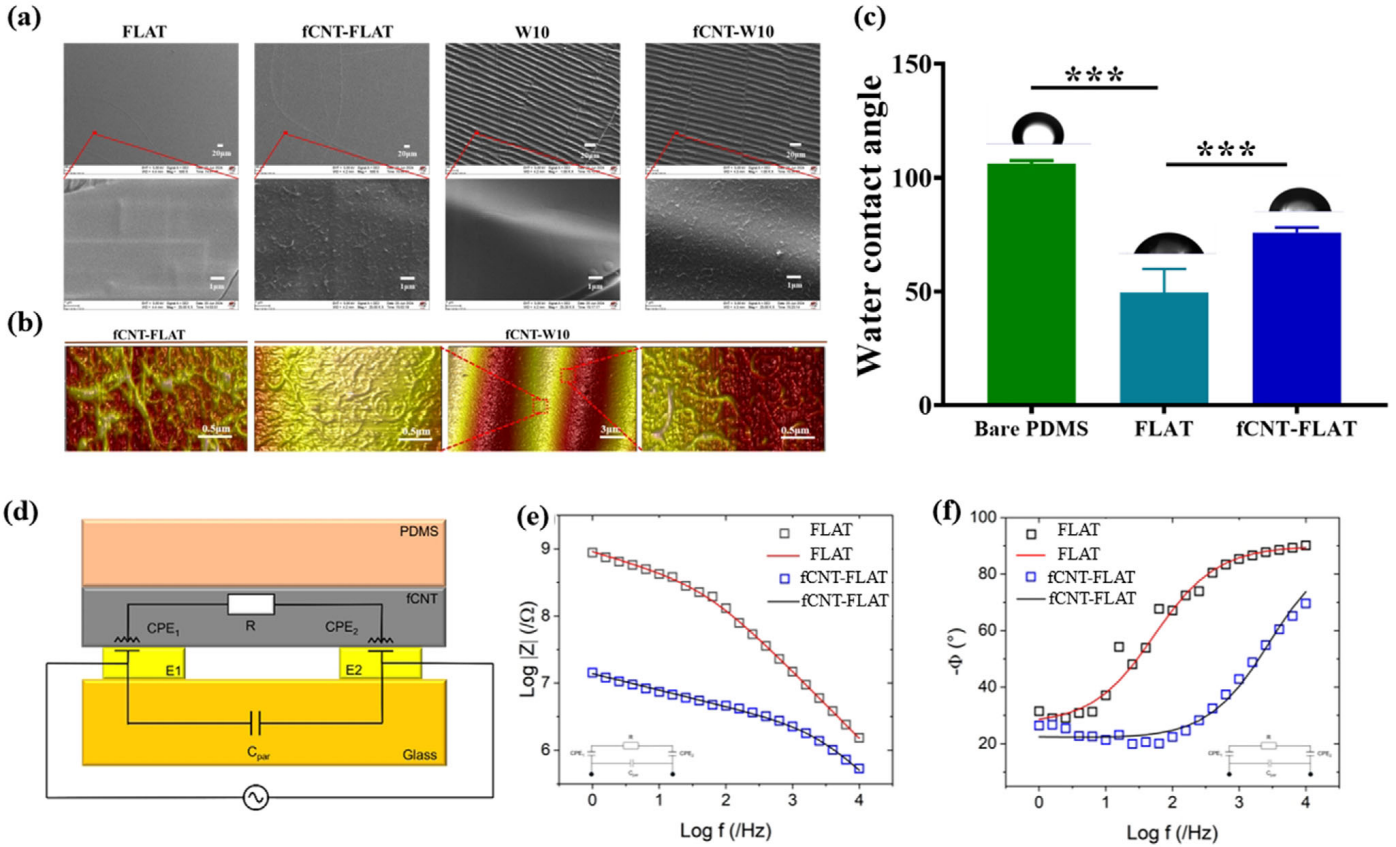
Surface property. a) SEM morphology of fCNT‐coated PDMS surface and without fCNT‐coated PDMS surface at low (up, 20 µm) and high (below, 1 µm) magnification. b) AFM images of fCNT‐coated PDMS surface and fCNT‐coated wrinkled PDMS surface, micro‐wrinkle size was 9.67 ± 0.32 µm in wavelength and 1.00 ± 0.08 µm in amplitude. c) Hydrophilicity property is measured by water contact angle on different surfaces. d) Schematic sketch of the PDMS cast on the microelectrodes and the corresponding equivalent circuit. Bode plots of the impedance modulus e) and phase f). Data are shown as mean ± standard deviation (SD) (***p < 0.001); three independent experiments were performed.

The fCNT coatings on Flat and wrinkled PDMS were further characterized by SEM and AFM. As shown in **Figure** **2**a,b, fCNTs were randomly distributed, recreating a uniform layer on the different surfaces and showing elongated morphology with nanometer‐sized fibers. In Figure S1 (Supporting Information), the morphology in the wavelength and the height of fCNT‐W10 showed a slight decrease because of the fCNT coating. And the difference between fCNT‐W10 and W10 was 0.078 ± 0.04 µm, which could be attributed to the thickness of the fCNT coating. The results indicated that fCNT will provide an increased nano‐scale surface structure.

The presence of a uniform coating was further exemplified by analyzing the hydrophilicity of the coating by water contact angle measurements. The insights for the surface polarity is best characterized on planar surfaces, at topography provides influences on the wettability using water contact angle measurements that are non‐existent in a completely wetted system. Therefore, we only measured the water contact angle on the FLAT sample rather than the wrinkled sample. As shown in Figure 2c. The contact angle was shown to be 76 ± 2° for fCNT‐FLAT films, while for FLAT uncoated (oxidized) PDMS, the water contact angle was 50 ± 10°, in contrast to non‐treated PDMS being 106 ± 1°. The changes in WCA indicate that the homogeneously coated PDMS has a lower WCA than non‐treated PDMS, but it is still moderately hydrophilic, even though the fCNTs are positively charged. The density will dictate in part how wettable the fCNT will be, and a lower density will result in more moderate wettability. The fact that the WCA changes before plasma activation and after coating indicates a good coverage of the surface with the fCNTs, which was also illustrated by the AFM analysis (Figure 2b). The alteration of the water contact angle illustrates the presence of the coating and the impact it has on the interaction with the environment, which includes protein adsorption. In fact, the fCNT‐coated surface demonstrated increased protein adsorption as compared to non‐coated PDMS, as shown in Figure S1 (Supporting Information).

One of the features that is suggested to facilitate interactions between myoblasts and scaffolds is conductivity.^[^ ^27^, ^28^ ^]^ To assess the electronic features of PDMS coated with fCNTs, we used impedance analysis. The FLAT and the corresponding fCNTs‐coated sample have been placed in contact with IDEs. The W10 and fCNT‐W10 could not be characterized, since their adhesion was too poor to establish an efficient electrical contact. Our system, FLAT and fCNT‐FLAT, is similar to the impedance cytometer presented by Guo et al,^[^ ^26^ ^]^ who exploited PDMS to separate the IDEs from the aqueous solution containing cells. The impedance data can be successfully fitted by using an equivalent circuit (Figure 2d) composed of a parasitic capacitance (C_par_) of the substrate, two constant phase elements that mimic the two electrode‐sample interface (CPE_1_ and CPE_2_) and the resistance (R) of either FLAT or fCNT (see Table S1, Supporting Information). Impedance modulus and phase show relevant discrepancies due to the presence of the fCNT (the corresponding Nyquist plot is shown in Figure S2, Supporting Information). The former shows a dramatic decrease featuring a lowering of two orders of magnitude from 100 up to 1 Hz. Moreover, the latter allows us to define even better the range wherein the fCNTs maximize their effect, namely close to 100 Hz. At this frequency, fCNT‐FLAT shows mostly a resistive behavior, whereas FLAT acts mostly as a capacitor. According to the resistive component of the two equivalent circuits, we extract two conductivities equal to 0.11 µScm^−1^ and 0.51 nScm^−1^ for fCNT‐FLAT and FLAT. Such conductivities are coherent with the modulus and phase trend that have been previously described, and they highlight the relevant impact of the deposition of fCNT onto the surface of PDMS.

### 2.2 Cell Viability, Proliferation, and Morphology on fCNT‐Coated Films

**Figure 3:**
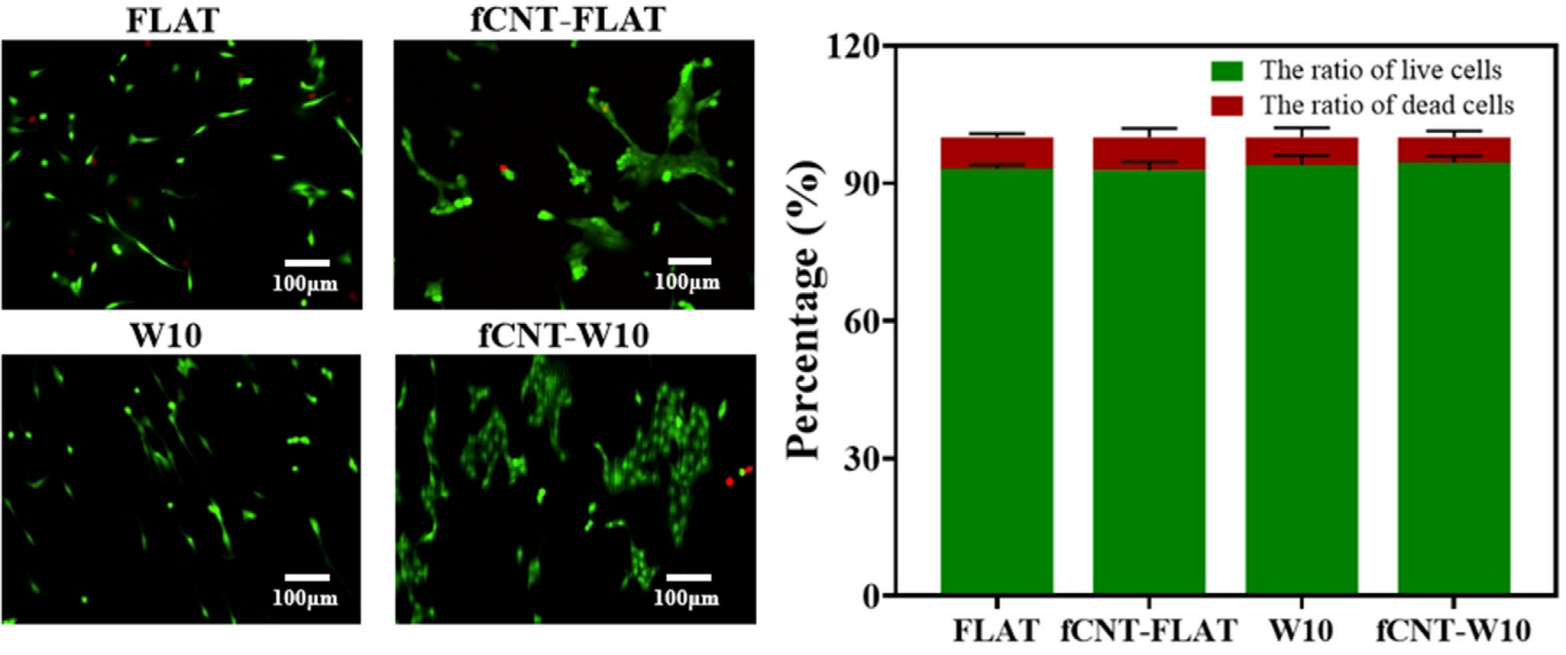
Live/Dead cell viability assay of V49 myoblast after 24 h cultured on fCNT‐coated surface and without fCNT‐coated surface. The cytoplasm of live cells emitted green fluorescence when stained with Calcein‐AM. The nuclei of dead cells emitted red fluorescence when stained with PI. The scale bar is 100 µm; 3 independent experiments were performed. Data are shown as mean ± standard deviation (SD). There is no significant difference between the groups.

To assess the cell viability and cytocompatibility of the fCNT‐coated samples, a Live/Dead assessment was performed (**Figure** **3**). V49 cells were seeded onto the substrate and allowed to adhere and grow for 24 h, and the viability was assessed. The data showed that the ratio of alive cells was above 90% in the different groups with no appreciable differences between them, reflecting that the fCNT‐coated materials had good cytocompatibility. Additionally, cell proliferation results shown in Figure S2 (Supporting Information) indicated that there was no significant difference among the different groups for 3 days by counting the number of nuclei, indicating that fCNT does not affect cellular proliferation.

**Figure 4:**
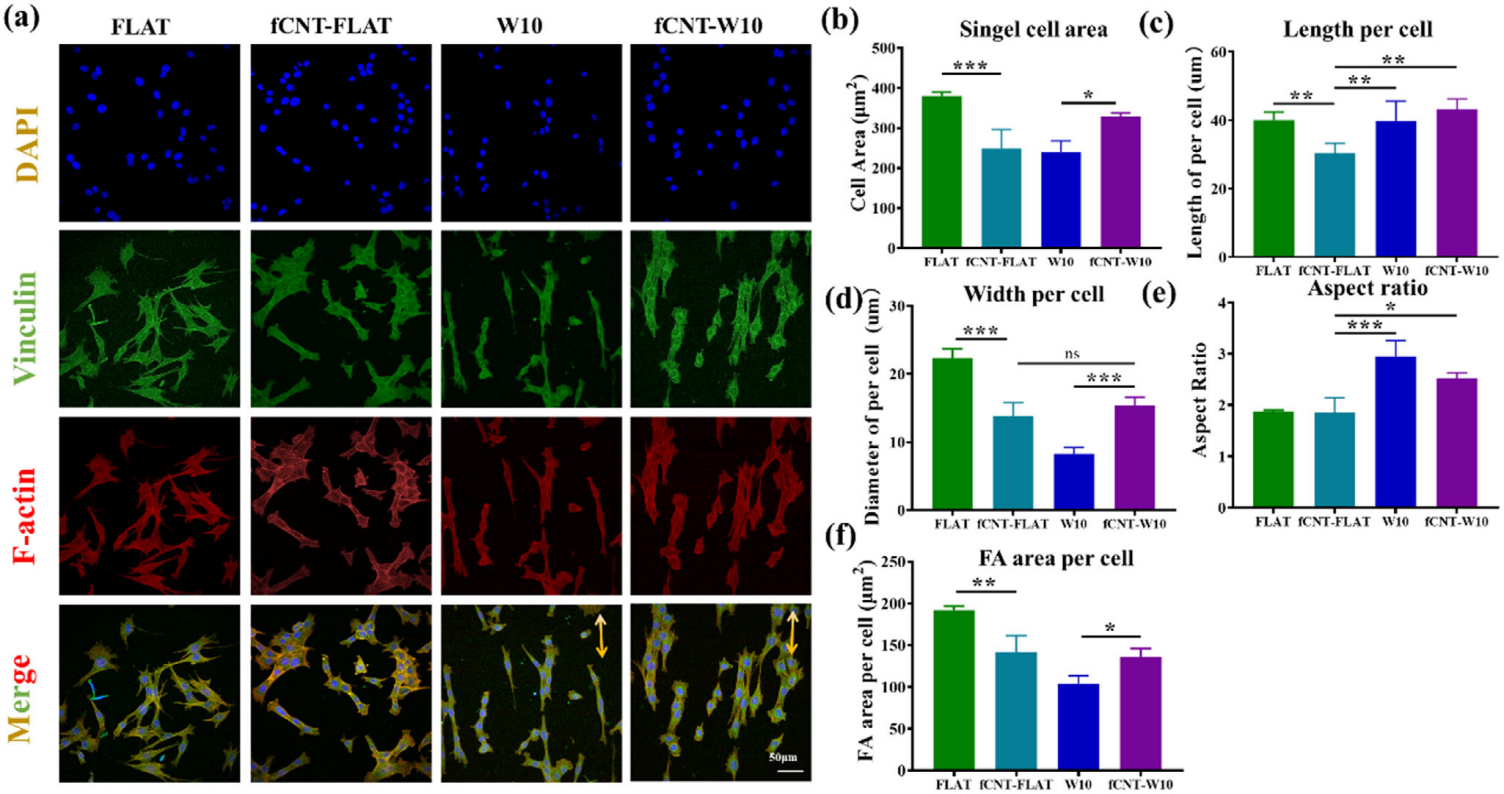
Cell morphology of myoblasts. a) Typical immuno‐fluorescence images of V49 myoblast after 24 h grown on TCP, fCNT‐coated surfaces, and without fCNT‐coated surfaces. Cell nuclei were stained with DAPI (blue), F‐actin was stained with TRITC‐labeled phalloidin (red), and vinculin was stained with green. b) Quantitative analysis of Area per cell, c) Cell length, d) Cell width, e)Cell aspect ratio, and (f) FA area per cell. The scale bar is 50 µm. Data are shown as mean ± standard deviation (SD) (*n* = at least 100 cells), *p < 0.05, **p < 0.01, ***p < 0.001). Three independent experiments were performed. The yellow solid arrows indicate the direction of the micro‐wrinkle surface.

Cellular behavior is mediated via a sensation known as contact guidance, spreading over the matrix surface with the filopodia extended to sense the surrounding microenvironment.^[^ ^27^ ^]^ Myoblasts were immunohistologically stained for nuclei, cytoskeleton, and vinculin to investigate detailed cell morphology and focal adhesion distribution. The fluorescence images were obtained by a confocal microscope after 24 h incubation. Differences in cell morphology and focal adhesion distribution are shown in **Figure** **4**a. The cells were aligned parallel to the wrinkled direction and adopted a more elongated morphology on wrinkled samples, while the cells grew randomly on FLAT and fCNT‐FLAT samples.

Following the observation from Figure 4b, the myoblasts on the FLAT had a larger cell spreading area than the fCNT‐FLAT sample, while the fCNT‐W10 group showed a larger cell area than the W10 group. This opposite influence of the fCNT on the cell on FLAT and W10 substrates is surprising. To explore the specifics of cell area changes, the cell length (Figure 4c) and cell width (Figure 4d) were quantified. The cell width was 13.82 ± 1.93 µm in the fCNT‐FLAT group, which was smaller than that of FLAT (22.33 ± 1.34 µm). Conversely, the cell width in the fCNT‐W10 group (15.39 ± 1.18 µm) was larger than that of the W10 group (8.28 ± 0.92 µm). Furthermore, the cell length was decreased with fCNT‐FLAT samples (30.36 ± 2.88 µm) compared with FLAT samples (40.00 ± 2.33 µm), while the wrinkled sample showed no significant difference, as illustrated by the quantitative data displayed in Figure 4c. These results demonstrate that the fCNT coating surface could alter the single cell spreading area, with the difference that fCNT limits the expansion of the cells in length and width on a flat surface, whereas fCNT increases the width of the cells without affecting the length on the wrinkle sample. Such influences could be a result of subtle interplays between different parameters that not only affect the overall morphology but also provide stimulation already on the subcellular scale, influencing the focal adhesions, as we also found in our previous works.^[^ ^28^ ^]^

The cell aspect ratio is the ratio of the major cell axis to the minor cell axis for a single cell; the higher the aspect ratio, indicates better the cell elongation.^[^ ^27^ ^]^ As shown in Figure 4e, FLAT and fCNT‐FLAT did not show significant differences and showed low elongation of cells compared to the wrinkled groups. However, the aspect ratio of the W10 group and fCNT‐W10 group were 2.95 ± 0.31 and 2.52 ± 0.11, respectively, which were larger than those on FLAT and fCNT‐FLAT samples. In addition to this, the combination of fluorescent images (Figure 4a) shows that the cells were aligned parallel to the wrinkled direction, while cells were randomly oriented on FLAT and fCNT‐FLAT samples. These results suggest that the microscale surface could regulate the cell elongation behavior and facilitate cell alignment, and that the fCNT coating influences V49 differently when surface topography is involved.^[^ ^7^ ^]^

Cell adhesion and spreading are essential for cellular communication and regulation, and mechanical interactions between cells and the underlying substrates can influence and control cellular behavior and functions.^[^ ^19^ ^]^ The focal adhesion (FA) structure was revealed by immunofluorescent staining of vinculin, which was distributed around the cells. To analyze the effects on the FA distribution of cells, we performed a quantitative analysis of FA area per cell, shown in Figure 4f. fCNT‐modified flat and wrinkled surfaces have different effects on myoblast cells. The fCNT reduced the FA area per cell for flat substrates, while for wrinkled substrates, the FA area per cell increased when fCNT was used. Compared to the cell area, the FA follows the same trends as the single‐cell area. These results indicated that the presence of fCNT had different effects on adhesion and morphology behavior in cells for Flat and wrinkled surfaces.

### 2.3 Myogenic Differentiation of Human Myoblasts on fCNT‐Coated Films

**Figure 5:**
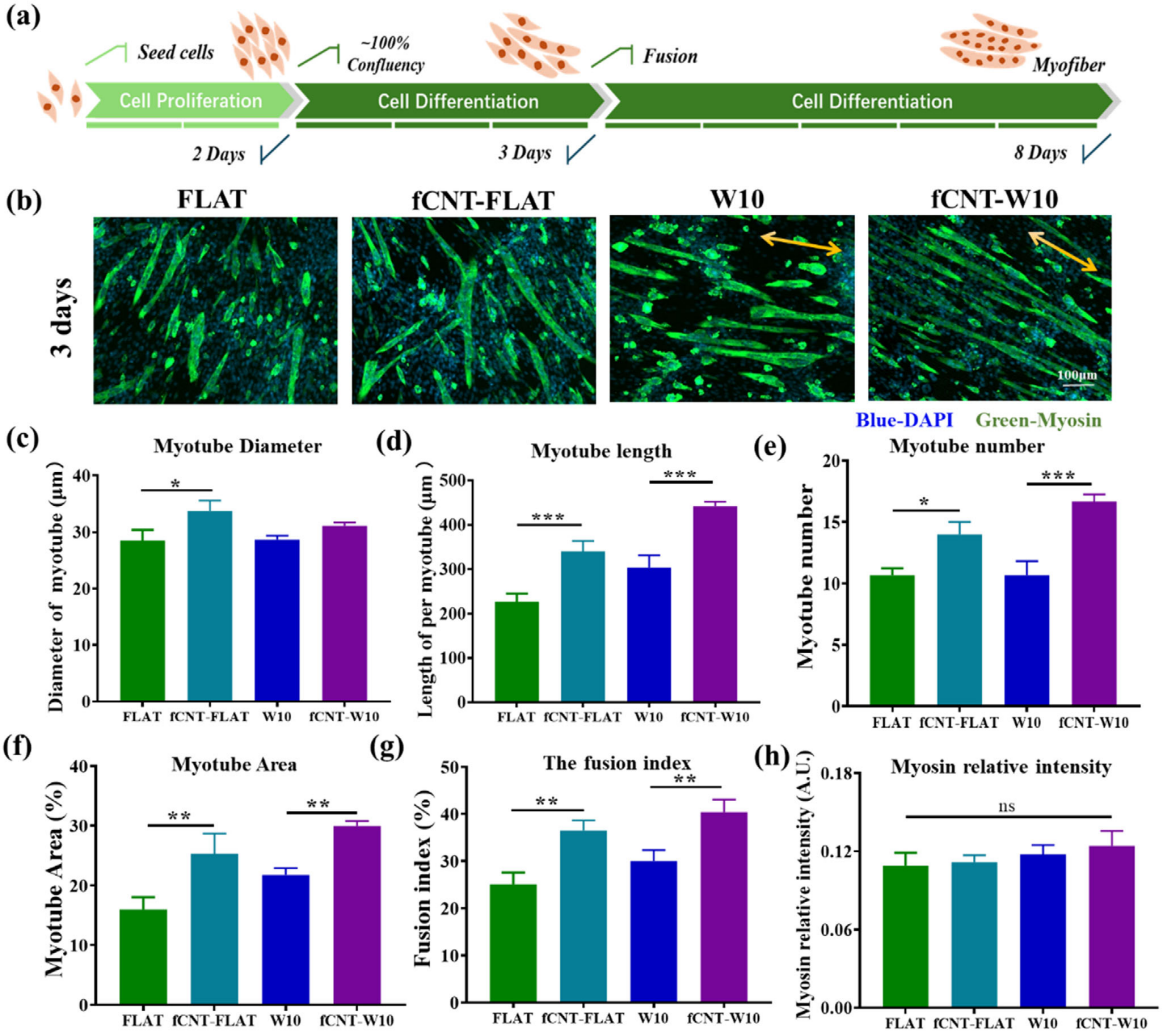
Fabrication of myotubes after 3 days of differentiation. a) Schematic illustration of the myogenic differentiation process. The cells were grown 2 days in proliferation medium, followed by starting to induce multiple myoblasts to fuse to myotubes, a multinucleated structure, in the presence of differentiation medium, and observed the fabrication of myotubes by staining nucleus and myosin heavy chain (a widely used myogenic differentiation marker) for 3 days and 8 days in differentiation medium. b) Typical immuno‐fluorescence images of V49 myoblast after 3 days of differentiation grown on TCP, fCNT‐coated surface, and without fCNT‐coated surface. The cell nuclei were stained with DAPI (blue), and Myotubes were stained with myosin heavy chain (green). c) Quantitative analysis of Myotube diameter, d) Myotube length, e)Myotube number, f) The total myotube area, g) The fusion index, which was calculated as the percentage of total nuclei inside the myotubes. h) Myosin fluorescence relative intensity normalized by the mean values of the total myotube area. The scale bar is 100 µm. Data are shown as mean ± standard deviation (SD) (*n* = at least 50 myotubes, *p < 0.05, **p < 0.01, ***p < 0.001); three independent experiments were performed. The yellow solid arrows indicate the direction of the micro‐wrinkle surface.

Myogenic differentiation toward multinucleated myoblasts is an essential phase of skeletal myogenesis. In the presence of low serum and induction medium, mononucleated myoblasts move and fuse to form multinucleated myotubes and reorganize their cytoskeleton as an initial building block for skeletal muscle growth and regeneration.^[^ ^29^, ^30^ ^]^ For the experiments, after 2 days of incubation, myoblasts were almost 100% confluent, which prevents cell density from affecting the myogenic differentiation process. To further determine the differentiation state of myoblasts, myotube morphology was stained with DAPI and myosin heavy chain antibodies on different substrates. Myosin heavy chain is the motor protein in muscle cells that drives the molecular processes of muscle contraction and confirms the differentiation.^[^ ^31^ ^]^ During the process, we examined the morphology of the formed myotubes induced by physically stimulated cells under differentiation conditions on the different substrates for 3 days (**Figure** **5**) and 8 days (Figure S4, Supporting Information).

In vivo, myofibers present a highly organized structure with extended parallel fascicles of multinuclear myotubes; Incorrect alignment of myotubes also leads to ineffective delivery of functional myofiber regeneration and contractile.^[^ ^1^ ^]^ So the myotube morphology, the degree of differentiation, and the ability of muscle contraction were assessed.

First, after 3 days of differentiation, multinucleated myotubes appeared on the different substrates. Myotube morphology was assessed through the immunofluorescence images and quantitative analysis of the myotube area, diameter, and length. As shown in Figure 5b, myotubes were prone to align when in contact with the wrinkle structure (Figure 5b), which is beneficial to imitate the aligned structure in vivo and provide support for normal muscle contractions. The diameter of myotubes shown in Figure 5c indicates that the fCNT‐coated groups promoted the thickness of myotubes on the fCNT‐FLAT surface, but not significantly on the fCNT‐W10 groups. For the length of the myotubes, in Figure 5d, the fCNT‐FLAT group (340.55 ± 23.32 µm) and fCNT‐W10 (441.63 ± 10.27 µm) showed a significant increase compared with the FLAT group (226.72 ± 18.29 µm) and W10 group (303.74 ± 27.61 µm). These results indicated that fCNT‐functionalized surfaces could guide cell differentiation and fusion to form wider, longer myotubes, an indication of increasing myotube maturity.

To further elucidate the cell differentiation capacity induced by the offered scaffolds, the myotube number and total myotube area were determined and are shown in Figure 5e,f. The fCNT‐FLAT and fCNT‐W10 groups enhanced the number of myotubes compared to FLAT and W10 (Figure 5e). At the same time, for both fCNT‐FLAT and fCNT‐W10 groups, the total myotube area was increased significantly to 25.31% ± 3.36% and 29.96% ± 0.83%, compared to their respective noncoated FLAT and W10, displaying 15.97% ± 2.07% and 21.74% ± 1.13%, respectively (Figure 5f). The results indicated that the fCNT‐coated sample promotes myotube formation during the differentiation process compared to non‐coated samples by observing myotube number and area. Besides, the fusion index reflected the myogenic index, the percentage of total nuclei inside the myotubes, which was one of the indicators of the degree of cell differentiation.^[^ ^32^ ^]^As shown in Figure 5g, the fusion index of fCNT‐FLAT increased to 36.50% ± 2.14%, and fCNT‐W10 groups enhanced to 40.43% ± 2.67%, which showed significant differences compared with those noncoated samples (the fusion index of FLAT group and W10 group were 25.13% ± 2.45% and 30.01% ± 2.34%, respectively.), indicating that fCNT‐coated groups showed excellent differentiation capacity.

Furthermore, to evaluate the capacity of muscle contraction, the fluorescence relative intensity analysis of myosin heavy chain molecular was analyzed in Figure 5h and showed no difference between the groups, indicating that myosin heavy chain molecular expression was not affected by the fCNT‐mediated substrates. These results revealed that micro‐scale surface modified with fCNT‐mediated nano‐scale surface could prompt myoblasts to form more myotubes, facilitate myotubes elongation, and accelerate the differentiation process in 3‐day differentiation.

In addition, since myotubes tend to delaminate under contraction processes and have poor adhesion property,^[^ ^33^ ^]^ to analyze the adhesion property between myotubes and substrates, 8 days of differentiation status was observed (Figure S4, Supporting Information) and we found that more myotubes remained and larger fusion index ratio was observed on fCNT‐coated samples compared to FLAT group and W10 group indicating that also for longer culture times the fCNTs have a positive influence. These results indicated that fCNT facilitates better adhesion. Therefore, fCNT‐W10 mediated surface could perform well on regulating myotubes alignment, elongation, and promoting myotubes formation in 3 days of differentiation, and showing more myotubes on the substrate for longer incubation, thereby promoting myotubes to form and mature larger myofibers.

## 3 Discussion

In this study, we used a multi‐walled CNT derivative (fCNT) modified with positively charged trimethyl ammonium benzene groups through a diazotization reaction^[^ ^23^ ^]^ to obtain a good dispersion in water. It was demonstrated that by applying a negative charge on the surface of PDMS by air‐plasma treatment and through electrostatic interactions, a stable fCNT coating was achieved. An effective and efficient coating method was established by dip coating, which provided a uniform fCNT coating with a combination of micro‐scale substrates for investigating the relationship between topography and cellular behavior stimulated with a conducting coating. The absence of complex processes and harsh conditions throughout the process makes the coating procedure much more reproducible, which is beneficial for further expanding production and the variety of different applications. The preparation of functional coatings on biological scaffolds is an important approach to improving their biocompatibility, mechanical properties, and bioactivity.^[^ ^1^ ^]^ Compared to existing methods (such as EPD, chemical grafting, and spray coating),^[^ ^34^ ^]^ our approach achieves a thinner CNT coating at a lower cost with uniform deposition. The modified scaffolds exhibited improved conductivity, optimized wettability, accelerated protein adsorption, good cell viability, enhanced cell response, and promoted cell differentiation, making them promising candidates for muscle tissue engineering applications. Mimicking and controlling cell‐topography interactions are pivotal for synthetic scaffolds.^[^ ^6^ ^]^ In our system, in contrast with individual microsize topography, fCNT offers an effective nano‐pattern, supplies electric conductivity, and presents an appropriate wettability, which could make it possible for muscle cells to grow on materials to produce and contract under electrical signals.^[^ ^15^, ^35^ ^]^ As tissue repair strategies advance, flexible biosensing devices capable of real‐time monitoring of scaffold performance can provide valuable insights into scaffold‐bioenvironment interactions.^[^ ^36^ ^]^ Conductive scaffolds are highly desirable for such applications, and this study offers an effective method for their preparation.

The fCNT coating increased the conductivity of the scaffold, rendering it capable of electrical stimulation, which could provide a new strategy for scaffold applications requiring electroactive properties. Furthermore, the fCNT coating optimized wettability and increased protein adsorption for the material surface, promoting cell attachment and spreading. The difference of water contact angle on different surfaces could be explained by the special nano‐sized structure and the charged fCNT surface.^[^ ^37^ ^]^ Besides, the surface surrounded by proteins derived from the biological environment is the first process in cell‐material interactions, occurring soon after introducing a biologically relevant liquid such as a culture medium upon implant insertion into the body.^[^ ^38^ ^]^ The dynamics of protein adsorption depend on both the special amphipathic structure of protein and the biomaterial properties, such as surface chemistry, charge, hydrophobicity, and shape by controlling the total amount of proteins bound to the surface, as well as their conformation and orientation after adsorption,^[^ ^37^, ^39^ ^]^ affecting the cellular performance of scaffold and implanted devices. As shown in Figure 2c and Figure S1 (Supporting Information), non‐plasma‐treated bare PDMS showed a hydrophobic surface, while the FLAT group showed hydrophilic properties. Combining the data of protein adsorption, nonoxidized PDMS, and FLAT (oxidized) exhibited similar values, which indicates that the difference in surface hydrophobicity and modification could affect the type of protein adsorption or its orientation at the surface without impacting the amount of protein adhered. The difference in protein adsorption in FLAT and W10 groups could be explained by the difference in specific surface area between FLAT and W10 groups; the specific surface area of W10 was larger due to the topography. Similarly, the ratio of surface area to volume of nano‐sized pattern provided by fCNT has increased, and the special fCNT surface causes the enhancement of protein adsorption amount.

Although the toxicity of fCNT cannot be ignored, macrophages can internalize free CNTs, which can lead to chronic inflammation and toxicity.^[^ ^40^ ^]^ While, in our study, the coating monolayer of fCNT by electrical interaction could effectively reduce multi‐walled CNT toxicity by using a small dosage and immobilizing them on the material surface, cells avoided the toxicity issues associated with free CNT exposure and had a high survival rate on fCNT‐coated surface as shown in viability results (Figure 3), making it possible to take advantage of electroactive multi‐walled CNT in a different range of tissue engineering applications. Furthermore, there was no significant difference among these groups for 3 days in cell proliferation, indicating that cells could maintain the proliferation property on the fCNT surface.

Satellite cells are the main drivers of skeletal muscle regeneration. A fine‐tuned balance between quiescent Satellite cells, activated to myoblasts, and differentiated states is a prerequisite for proper regeneration.^[^ ^41^ ^]^ The reorganization of focal contacts and intracellular cytoskeleton organization is essential for cell communication and regulation via mechanical interactions between cells and underlying substrates.^[^ ^42^ ^]^ The remodeling of the actin cytoskeleton and the formation of integrin clusters associated factors, activation of FAK/ERK^[^ ^43^ ^]^ and RhoA/ROCK signal pathway,^[^ ^41^, ^44^ ^]^ and the regulation of MicroRNA^[^ ^45^, ^46^ ^]^ modulate cell behavior. For adherent cells, cell motility presupposes attachment of the cells. The area of contact between the cell and the substrate determines cellular further motility.^[^ ^47^, ^48^, ^49^ ^]^ The tubular‐sized fCNT could significantly influence cell morphology and try to regulate and control cells to grow into a certain size as shown in Fig, 4. The interaction in cell morphological transition on fCNT can be explained in terms of the tubular nano‐size pattern and the distribution of the adsorption of serum and cell‐secreted adhesive proteins to the nanotubes. It has been reported that nano‐sized CNT could activate cellular nanoscale sensing mechanisms by corresponding to integrin clustering, and the cell attachment and spreading were integrin‐dependent via recognizing adhesive proteins (like fibronectin and collagen IV) on the nanotubes.^[^ ^19^, ^50^ ^]^ Many studies have taken advantage of CNT to improve the bio‐adhesion of materials in 3D structures and regular stem cell fate.^[^ ^42^, ^51^ ^]^ The changes in interfacial physicochemical factors affect cell morphology and behavior, such as changes in surface pattern, hydrophilicity, protein adsorption, and protein species. Here, we do not know exactly which factor plays a dominant role or the synergistic effect of multiple factors. The interactions between material surfaces and cells are sometimes not what we expect, and instead of cells showing the expected linear correlation change as a single variable is altered, synergistic interactions between different physicochemical parameters can affect changes in the morphology and behavior of the cells.^[^ ^35^, ^52^, ^53^ ^]^

Studies have already proved that electrical stimulation created by the subcellular level highly impacts myogenesis which could accelerate the assembly of sarcomeres and support muscle maturation by intracellular calcium signaling (Ca^2^⁺‐dependent pathways (NFAT, CaMKII)),^[^ ^12^, ^54^ ^]^ it can alter membrane potential to trigger calcium influx through voltage‐gated L‐type calcium channels.^[^ ^13^, ^14^, ^15^ ^]^ Zhao et al. have proved that PEG‐CNT films could direct the skeletal myogenic differentiation of hMSCs in the absence of myogenic induction factors.^[^ ^55^ ^]^ And the existing studies have demonstrated that aligned nano‐topography and conductive materials can enhance myoblast differentiation, with the degree of myogenic differentiation increasing with higher material conductivity.^[^ ^56^, ^57^, ^58^ ^]^ In our study, the electroactive fCNT enhanced myoblast differentiation, which probably involved ion flow, and induced elevated levels of intracellular calcium ions. In addition, the native skeletal muscle exhibits a highly organized structure with extended alignment arrays of multinucleated myofibers, and many studies confirmed how important the directional myotube arrangement is, especially in the regeneration of functioning muscle fibers for effective force transmission and contractility.^[^ ^7^, ^59^ ^]^ Besides, the combination of nano and microscale topological surfaces had already been verified to enhance myogenic differentiation by increasing the expression of both early and late‐stage myogenic differentiation genes associated with myogenic and myosin heavy chain.^[^ ^60^ ^]^

Overall, we hypothesize that the acceleration of myogenesis differentiation and myotube formation in our system could depend on the synergy of micro‐/macroscale structures, the surface physicochemical property, and electroactive fCNT and while in many cases it has been demonstrated that CNTs induce positive results on many electro‐active tissues,^[^ ^61^ ^]^ the contributions of the altered charge and chemical compositions should be noticed as these can also have a stimulating or inhibiting effect and hence here we look at a combined influence of the applied coating. Although more in‐depth studies are needed to elucidate the mechanistic events of this kind of synergistic effect in the accelerated myotube formation on fCNT‐coated surfaces as well as identifying to what extent the CNT parameters themselves (length, diameter, multi‐walled/single walled, conductivity) might influence the cellular process, the results demonstrated here support the efficacy of fCNT‐coated surfaces in modulating the myoblasts differentiation process, thus highlighting their potential as conductive substrates for muscle tissue engineering.

## 4 Conclusion

Here we reported the fabrication and characterization of fCNT‐coated films to support myoblasts' growth and differentiation. The fCNT‐W10 substrate could present special topography, optimized wettability, and improved electrical conductivity properties. It is interesting to find that the fCNT‐coated film triggered different cell morphology on flat and micro‐sized wrinkled surfaces. Meanwhile, cell differentiation was enhanced by fCNT‐coated film, and myotube orientation was induced on an fCNT‐W10 substrate to better represent the natural state of the tissue. These collective findings support that the fCNT‐coated surface combined with micro‐sized structure is a promising way for muscle growth, making them promising candidates for muscle tissue engineering applications.

## 5 Experimental Section

### Preparation of fCNT‐Coated PDMS Substrate

*Aligned surface topography formation*: The PDMS films were fabricated by mixing the elastomer and cross‐linker in 1:10 w/w ratio (SYLGARD 184 Silicone Elastomer Kit, Dow Corning). The mixtures were deposited onto a 12 × 12 cm petri dish and cured in an oven at 70 °C for 3 h. After curing, the PDMS was cut into 9 × 9 cm. The wrinkled PDMS films were prepared by the plasma treatment technique and imprinting method to obtain the final aligned wrinkled PDMS, as described previously.^[^ ^6^ ^]^ Flat PDMS was stretched by a stretching apparatus to 20% of the original length and subsequently treated with plasma (Plasma Activate Flecto 10 USB) for 10 min at 25 mTorr. The stress was removed, which induced wrinkle formation with 9.67 ± 0.32 µm in wavelength and 1.00 ± 0.08 µm in amplitude. To guarantee reproducibility, we fabricated wrinkles by using a mould to produce more uniform wrinkled PDMS substrates. For this, the first wrinkled PDMS substrate was treated with plasma for 10 min at 100–150 mTorr by air‐plasma to obtain a uniformly treated surface and waited for one week in air to get a hydrophobic surface that was subsequently used as a mold. On the mold, fresh pre‐gel solution (mixing the elastomer and cross‐linker in 1:10 w/w ratio) was poured, followed by curing at 70 °C for 3 h. The molds were removed, and the fresh wrinkled PDMS substrates were prepared.

*Synthesis of ammonium‐functionalized multi‐walled CNTs (fCNT)*: multi‐walled CNT (ACS Material, Pasadena, CA, USA; OD 8–15 nm, l. 0.5–2 µm) was functionalized with N,N,N‐trimethylbenzene ammonium groups as described previously.^[^ ^23^ ^]^Briefly, multi‐walled CNTs (70.0 mg, 5.82 mmol of C) was mixed with a solution of 4‐amino‐N,N,N‐trimethylbenzene ammonium iodide^[^ ^62^ ^]^ (810.51 mg, 2.91 mmol) and isopentyl nitrite (0.824 mL of 95% Sigma‐Aldrich, 5.82 mmol) at 80 °C under nitrogen flux and magnetic stirring. After 4 h, the reaction mixture was allowed to cool at room temperature, and the product was recovered by filtration on a Millipore PC 0.1 µm membrane (VCTP) and washed through six subsequent sonication and filtration cycles with distilled water and methanol. The methanol dispersion was finally dried under nitrogen flux to afford the multi‐walled CNT derivative (fCNT) as a solid black powder.

*fCNT‐Coating formation*: A fCNT solution (1 mg mL^−1^) was prepared in MilliQ water by sonicating for 5 min with a tip sonicator to obtain a homogeneous dispersion. The PDMS slides were cleaned with 70% ethanol, followed by MiliQ water, and dried with pressured air. To deposit fCNT on the PDMS substrate, the PDMS films were first oxidized with air‐plasma for 10 min at 100–150 mTorr to obtain a negatively charged surface, followed by either 1) spraying the solution of positive charged fCNT onto the oxidized PDMS substrates until the PDMS surface was completely wet with subsequent drying at air overnight (Figure 1a) or 2) by immersing the oxidized PDMS slides into the solution of positively charged fCNT overnight as a dip coating method (Figure 1c). During this procedure, the oxidized PDMS was turned over and floated in the fCNT suspension, which allowed full contact between the negatively charged surface and the fCNT suspension, avoiding fCNT sediment on the surface and forming an uneven coating surface. Next, the treated PDMS substrates prepared by the above methods were carefully immersed in MilliQ water for 6 h to remove excess fCNT and were replaced the MilliQ water every 2 h to obtain a homogeneous fCNT‐coated layer on the PDMS surface. The flat and wrinkled PDMS coated with fCNT is designated as “fCNT‐FLAT” and “fCNT‐W10”, respectively, and pure plasma‐treated flat and wrinkled PDMS as “FLAT” and “W10”, respectively.

SEM analysis was performed on the surfaces of the samples by sputtering a thin chromium layer of 5 nm. SEM images were acquired with a Zeiss Sigma HD microscope, equipped with a Schottky FEG source and a backscattered electron detector. Analysis was performed in a high vacuum at 2 × 10^−5^ mbar.

For a more detailed investigation of the nano‐scale morphology of fCNT on the PDMS surface, the fCNT‐coated PDMS surfaces were analyzed by Atomic Force Microscope (NanoScope V AFM, Bruker, Billerica, MA, USA) operating in tapping mode in air. A Bruker model DNP‐10 tip of nonconductive silicon nitride was used for measurement (in air mold). The morphology of the surface in the images was analyzed by NanoScope Analysis software (Bruker).

### Water Contact Angle Measurements

The samples were prepared and dried with pressurized air, and the water contact angle measurement was done immediately by an in‐house developed tensiometer. The static contact angle measurement was determined using the sessile drop method. Droplets of ≈5 µL of MilliQ water were placed on the surfaces, and the images of the droplets were analyzed for contact angles. Bare PDMS represents fresh PDMS without plasma treatment. Three independent imprints were analyzed, and at least five images were analyzed for each substrate.

### Protein Adsorption

The samples were cut into circular disks matching the diameter of a well in a 96‐well plate, 200 µL of a 1% FBS was added to the different samples and incubated for 30 min at 37 °C after which the FBS solution was removed. Protein adsorption was measured by BCA protein assay kit (Thermo Fisher, USA). Briefly, 200 µL work solution of BCA reagents A and B with the ratio of 20:1 was added to the FBS‐treated substrates within the 96‐well plate and incubated for 30 min at 37 °C. The absorption of all the samples was recorded by a microplate reader (Bio‐Rad, USA) at 562 nm. The protein adsorption value was calculated using the following equation:

(1) ODvalue=ODtest−ODblank

where OD test and OD control were the absorbance of the tested sample (samples were incubated with BSA) and the control sample (samples without incubating with BSA solution). The experiments were performed in triplicate.

### Conductivity Measurement

The impedance analysis has been performed by using interdigitated electrodes (IDEs) on a glass substrate (G‐IDE222 purchased from DropSens, Metrohm). More specifically, IDEs were composed of 52 pairs of digits, 10 µm wide and equally spaced (viz. 10 µm). The total area is equal to 4 mm^2^, on the contrary, the area in between the interdigitated electrodes is equal to 1.75 mm^2^ and the meandering path is 17.47 cm long. The electrochemical cell is composed of these two IDEs, thereby acting as working and counter electrodes. Since PDMS is an insulator, we applied a setpoint potential equal to 100 mV in order to acquire a reliable signal spanning from 10^4^ and 10^−1^ Hz.

The whole batch of electrochemical measurements was performed by placing the samples, FLAT and fCNT‐FLAT, in direct contact with the IDEs, and the impedance data were acquired by using a Metrohm Autolab Potentiostat/Galvanostat PGSTAT302N.

### Cell Culture

The human myoblasts (V49 satellite cells) were kindly provided by Prof. Dr. Marco Harmsen and displayed high self‐renewal and cloning capacity, which was the same as those used in our previous study.^[^ ^7^, ^63^ ^]^ The general culture conditions are as follows: myoblasts were cultured in proliferation medium (PM), comprised of DMEM‐HG (Gibco) supplemented with 20% FBS (Gibco) and 1% p/s (Gibco) under 5% CO^2^ and 37 °C. For the differentiation stage, upon reaching confluence, the medium was changed to differentiation medium (DM), comprised of DMEM‐HG, 1% FBS, 1% p/s, 1% Insulin‐Transferrin‐Selenium (Gibco), and 1% dexamethasone (Sigma‐Aldrich). Clone V49 was used between Passages 10 and 13. The myoblasts were seeded into the FLAT, fCNT‐FLAT, W10, fCNT‐W10, which were cut into circular disks matching the diameter of a 24‐well plate. All samples were treated with 70% ethanol for sterilization and washed with PBS before use.

### Cell Viability

Myoblasts were seeded at a density of 4 × 10^4^ cells and cultured in PM solution to allow the free spread for 24 h. The viability of cells on different substrates was determined by using Live/Dead staining (Sigma‐Aldrich). Quantification of live cells (green) and dead cells (red) by counting the number of live cells and dead cells in Fiji software. Three independent experiments were performed (*n* ≥ 5).

(2) $$\mathrm{Cell}\;\mathrm{viability}\left(\%\right)=\mathrm{live}\left(\mathrm{dead}\right)\mathrm{cells}\;\mathrm{number}/\mathrm{Overall}\;\mathrm{cell}\;\mathrm{number}\times 100\%$$

### Cell Morphology

The myoblasts were seeded at a density of 2 × 10^4^ cells and cultured in PM solution to allow them to spread for 24 h. Afterward, the cells were first washed with PBS and fixed using 3.7% paraformaldehyde (Sigma‐Aldrich) for 15 min, then the cell membrane was permeabilized with 0.5% Triton X‐100 solution for 15 min, followed by blocking with 5% BSA in PBS for 30 min. Subsequently, substrates were incubated with vinculin (V9131, Merk, 1:100) as primary antibody for 3 h at 4 °C, followed by incubating with Goat Anti‐Mouse IgG H&L (Alexa Fluor 488) (ab150113, Abcam, 1:500), Tritc‐phalliodin (P1951, Sigma‐Aldrich) and DAPI in PBS with 1% BSA for 1 h. Immunofluorescence imaging was done by a confocal microscope (Microscope Leica Stellaris 5). Quantification of focal adhesion by vinculin and the cell cytoskeleton was performed by Fiji software. Three independent experiments were performed.

### Cell Proliferation

The myoblasts were seeded at a density of 2 × 10^4^ cells and cultured in PM solution to allow the to freely spread overnight. After which the cells were incubated for 24, 48, and 72 h. Cell proliferation was measured by counting cell nuclei using Fiji software. The cells were first washed with PBS and fixed using 3.7% paraformaldehyde (Sigma) for 15 min, then the cell membrane was permeabilized with 0.5% Triton X‐100 solution for 15 min, followed by incubating with DAPI. Three independent experiments were performed.

### Cell Differentiation

The myoblasts were seeded at a density of 8 × 10^4^ cells after 3 days of proliferation in PM solution to 100% confluence, followed by changing to DM solution and fixing cells for 3 days and 8 days for immunostaining. For staining protocol, the cells were fixed, permeabilized and incubated with the primary antibody for mouse‐anti‐human Myosin heavy chain (Myosin 4 Monoclonal Antibody (MF20), Thermofisher, 1:200) in PBS with 1% BSA for 3 h at 4 °C, then incubated with Goat Anti‐Mouse IgG H&L (Alexa Fluor 488, 1:500) in PBS with 1% BSA and DAPI for 1 h. Immunofluorescence imaging was done by a fluorescence microscope (Microscope DM4000b). Myotube area, diameter, length, and expression were evaluated by Fiji software. CellProfiler evaluated the fusion index. For each substrate, at least 15 images were analyzed. Three independent experiments were performed.

### Statistical Analysis

All data were expressed as mean value ± standard deviation. Statistical analysis was performed with GraphPad Prism 6 software. All data were carried out by one‐way analysis of variance (ANOVA), with a *p*‐value (<0.05) considered statistically significant.

## Conflict of Interest

The authors declare the following financial interests/personal relationships which may be considered as potential competing interests: Patrick van Rijn reports a relationship with BiomACS BV that includes equity or stocks. P.V.R. is also the co‐founder, scientific advisor, and shareholder of BiomACS BV, a biomedical‐oriented screening company. The authors declare no other competing interests. The authors declare no further conflict of interest.

## Supporting information



Supporting Information

## Data Availability

The data that support the findings of this study are available from the corresponding author upon reasonable request.
