# Supplementary material for: Functionalized Multi‐Walled Carbon Nanotube Enhanced Myogenic Differentiation for Aligned Topography‐Induced Skeletal Muscle Engineering
Source: Small. 2025 Jul 24;21(35):2504992. doi: 10.1002/smll.202504992 (PMC12410905; doi:10.1002/smll.202504992)
Supplement: Supplementary file 1 — Supporting Information [file SMLL-21-2504992-s001.docx]

**Functionalized multi-walled carbon nanotube enhanced myogenic differentiation for aligned topography-induced skeletal muscle engineering**

***Tianqi Feng^a^****^,#^****, Ludovica Ceroni^b^****^,#^****, Lisa Eveline Tromp^a^, Clio Siebenmorgen^a^, Stefano Casalini^b^, Enzo Menna^b,^*, Patrick van Rijn^a,^****

**Supporting information**


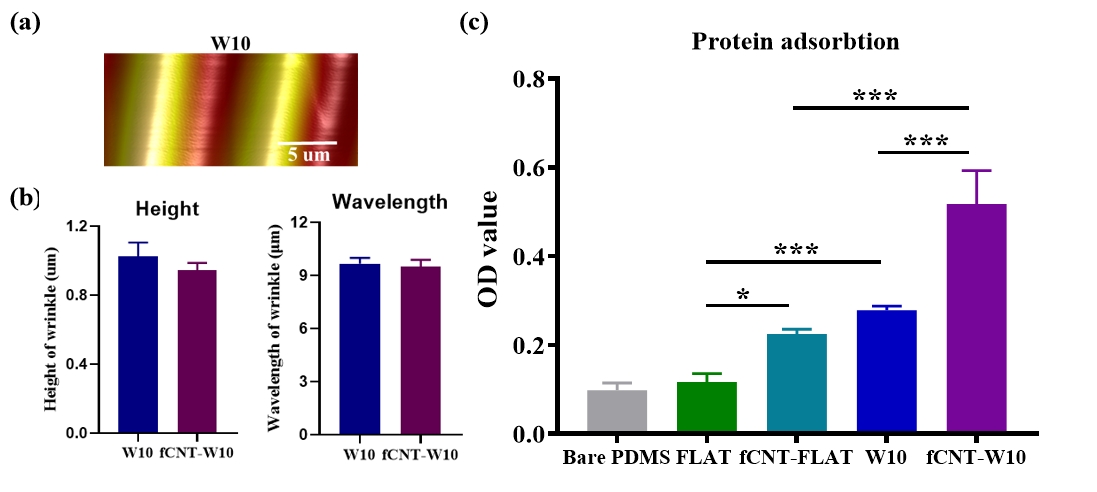


**Figure S1.** (a) AFM image of W10 group. (b) The wavelength and height of W10 and fCNT-W10 groups. (c) The Protein absorption on Bare PDMS(without plasma-treated PDMS), fCNT-coated surface, and without fCNT-coated surface. The absorbance intensity (at 562 nm) was generated during the incubation of BCA working solution with 1 % FBS solution. Data are shown as mean ± standard deviation (SD) (*P< 0.05, **P<0.01, ***P<0.001).

**Table S1** Values of the different element composing the equivalent circuit. R, Cpar and CPE1,2 are the resistance of the material of interest, the capacitance of the substrate and the constant phase elements of the two interfaces of the material-microelectrodes.

| Type of substrate | R (Ohm) | C_par_ (F) | CPE_1,2_ (S∙s^n^) |
| --- | --- | --- | --- |
| FLAT PDMS | 1.12∙10^8^ | 1.05∙10^-11^ | 6.62∙10^-10^ n = 0.32 |
| fCNT-FLAT PDMS | 5.22∙10^5^ | 2.76∙10^-11^ | 4.65∙10^-8^ n = 0.26 |


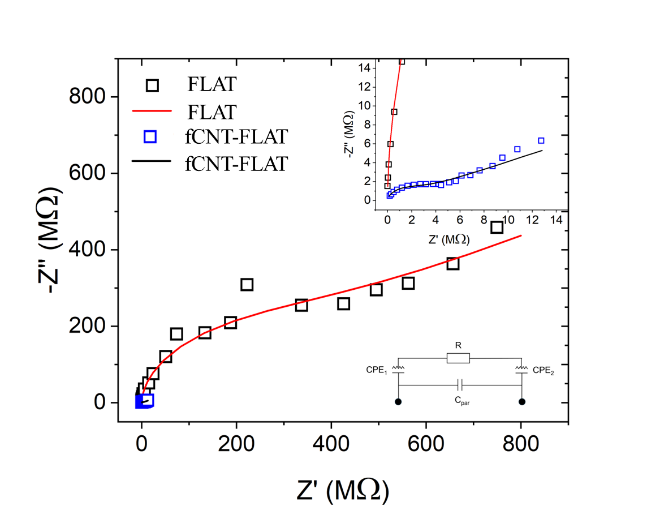


**Figure S2.** Nyquist plot overlay of the two samples: FLAT PDMS (empty black squares) and fCNT-FLAT PDMS (empty blue squares).


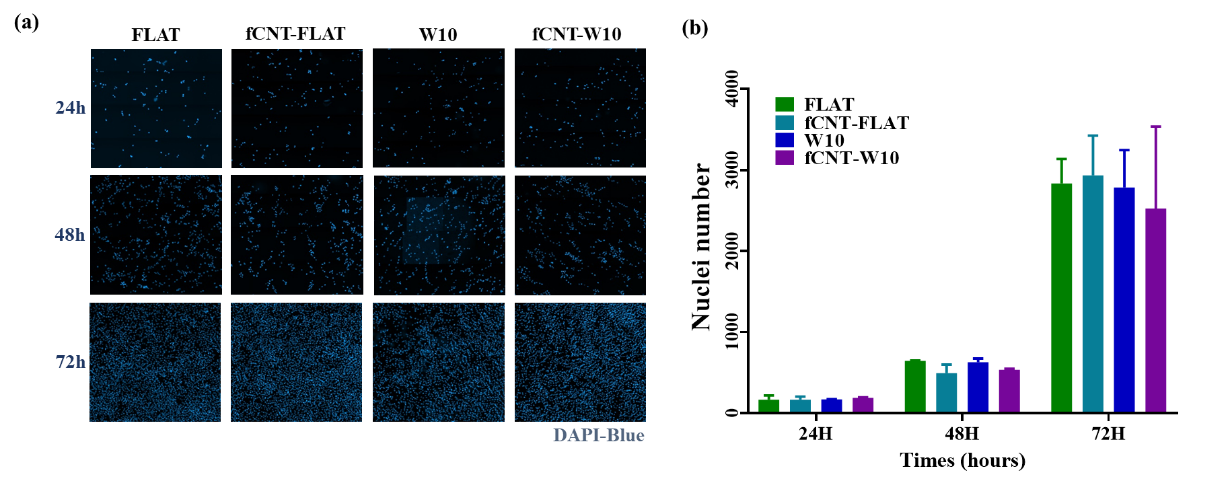


**Figure S3.** Myoblast proliferation. (a) Typical immuno-fluorescence images of V49 myoblast after 24 h, 48 h, and 72 h proliferation grown on fCNT-coated surface, and without fCNT-coated surface. The cell nuclei were stained with DAPI (blue). (b)The nuclei numbers. The data were shown as mean ± standard deviation (SD).


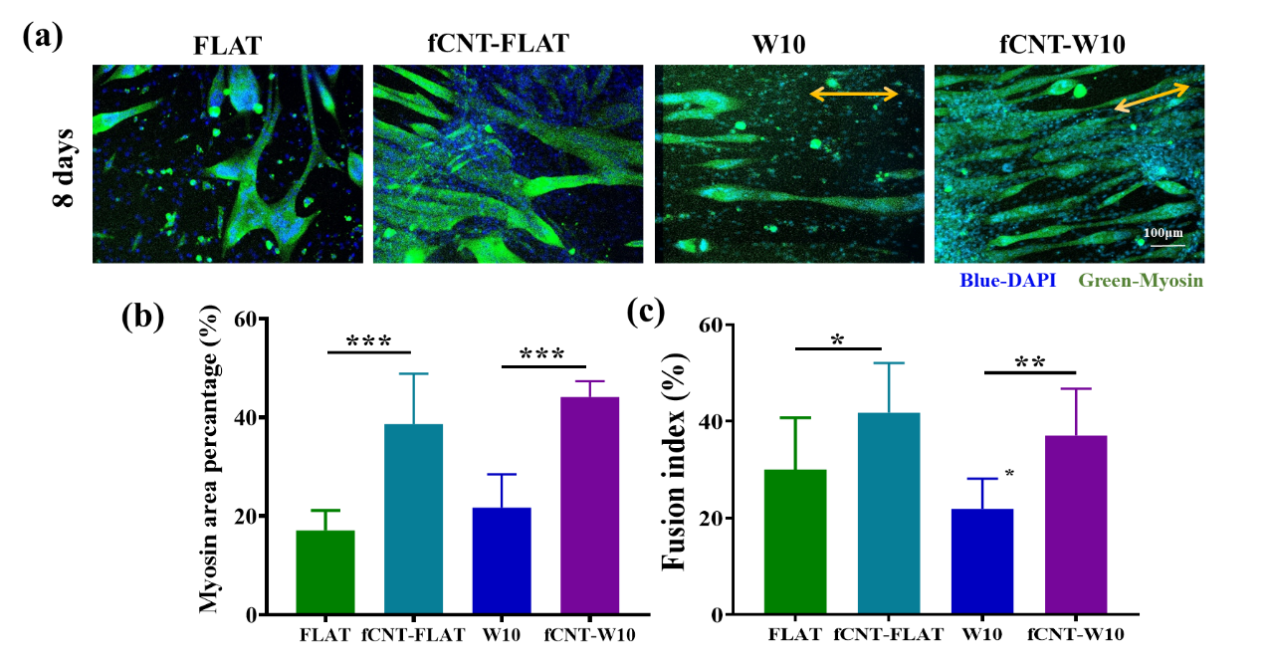


**Figure S4.** Fabrication of myotubes after 8 days of differentiation. (a) Typical immuno-fluorescence images of V49 myoblast after 8 days differentiation grown on fCNT-coated surface, and without fCNT-coated surface. The cell nuclei were stained with DAPI (blue) and Myotube was stained with myosin heavy chain (green). (b)The total myotube area. (c) The fusion index. The scale bar is 100 μm. Data are shown as mean ± standard deviation (SD) (n=at least 50, *P< 0.05, **P<0.01, ***P<0.001). The yellow solid arrows indicate the direction of the micro-wrinkle surface.
